# Transperineal anastomotic posterior urethroplasty with bulbocavernosus flap and fibrin sealant in the complicated posterior urethral stricture reconstruction: a retrospective cohort study

**DOI:** 10.1097/JS9.0000000000000890

**Published:** 2023-11-16

**Authors:** Menghua Wang, Liang Zhou, Banghua Liao, Donghui Ye, Yucheng Ma, Zhongyu Jian, Chi Yuan, Xi Jin, Hong Li, Kunjie Wang

**Affiliations:** a Department of Urology, Institute of Urology (Laboratory of Reconstructive Urology), West China Hospital; b West China Biomedical Big Data Center, Sichuan University, Chengdu, Sichuan; c Department of Urology, The First Affiliated Hospital, Zhejiang University School of Medicine, Hangzhou, People’s Republic of China

**Keywords:** bulbocavernosus flap, fibrin sealant, urethral stricture, urethroplasty

## Abstract

**Background:**

Management of complicated posterior urethral stricture is challenging. Modified transperineal anastomotic urethroplasty (TAU) with bulbocavernosus flap interposition and human fibrin sealant provides another treatment option. The authors aimed to evaluate whether this technique could improve the success rate in the complicated posterior urethral stricture reconstruction in this study.

**Materials and methods:**

Between 2016 and 2019, 48 patients underwent either conventional or modified TAU. The criteria for success included both the absence of clinical symptoms and no need for further surgical intervention during follow-up.

**Results:**

Twelve patients underwent the modified TAU (group A) using bulbocavernosus flap interposition and human fibrin sealant. Thirty-six patients underwent the traditional end-to-end anastomotic urethroplasty (group B). Follow-up was 24.3–57.2 months. The patients in group A had a higher surgery success rate compared to the patients in group B (91.7 vs. 63.9%, *P*=0.067), with a quasi-significant result. Besides, no postoperative complications were observed in group A, while two individuals in group B had urinary incontinence, but the difference was not significant (0 vs. 5.6%, *P*=0.404).

**Conclusion:**

Based on the preliminary results, modified TAU with bulbocavernosus flap interposition and human fibrin sealant is a safe and feasible technique for complicated posterior urethral stricture reconstruction.

## Introduction

Highlights

- Management of complicated posterior urethral stricture is challenging.
- We reported a novel technique for complicated posterior urethral stricture reconstruction, which is safe and feasible based on our preliminary results.
- Further well-designed prospective trials with a larger sample size are necessary to obtain conclusive results.

Urethra stricture is one of the most common urological diseases and can be classified into anterior and posterior urethral stricture^1^. If left untreated, it might cause long-term damage to the entire urinary tract, like elevated voiding pressure and bladder fibrosis^2^. The primary recommended treatment for posterior urethral stricture is suprapubic cystostomy, followed by delayed transperineal anastomotic urethroplasty (TAU)^3,4^. The mean success rate of TAU is about 80%^5^. However, for the complicated posterior urethral strictures with long-segment strictures^6^ or previously failed surgical repair, conventional TAU might not be perfect, since the big dead space caused by intraoperative long-segment scar resection may increase the incidence of recurrence and postoperative complications like infection and fistula.

In our center, two techniques were used to eliminate the dead space, which were bulbocavernosus flap interposition and fibrin sealant injection. The bulbocavernosus flap is a natural and excellent interposition material to fill the dead space. Additionally, the nature of fibrin sealant could further promote hemostasis and tissue adhesion. These two techniques can complement each other to fill the dead space and might improve the success rate.

In this research, we would like to assess whether this modified TAU with bulbocavernosus flap and human-plasma-derived fibrin sealant could improve surgery success rate in the complicated posterior urethral stricture reconstruction.

## Material and methods

This work has been reported in line with the strengthening the reporting of cohort, cross-sectional and case–control studies in surgery (STROCSS) criteria^7^.

### Study population

We screened our institutional medical records to identify all the male patients with complicated posterior urethral stricture from 2016 to 2019. Complicated posterior urethral stricture was defined as an intraoperatively measured gap exceeding 2 cm or the previously failed repair surgery. The etiology of urethral strictures among all the eligible patients were pelvic fracture. Those patients with urethral rectal fistula, bladder neck injury or false passage would be excluded. According to the type of surgery, patients were divided into group A (undergoing the modified TAU with bulbocavernosus flap interposition and human fibrin sealant) and group B (undergoing the conventional TAU).

To balance the covariates distribution between group A and group B, we conducted 1:3 nearest-neighbor propensity score analysis in our study, which was based on all the baseline characteristics in Table 1. One covariate that needs special explanation is the relative location between the proximal urethra end and pubic ramus, which has been identified as an essential predictor for prognosis in our prior study^8^.

**Table 1:** Baseline characteristics and results of included patients.

	Modified TAU (*n*=12)	Traditional TAU (*n*=36)	*P*
Baseline Characteristics			
Age (years)	38.7±14.5	41.2±12.7	0.559
BMI (kg/m^2^)	21.1±4.5	21.6±2.6	0.754
Duration between suprapubic cystostomy onset and urethroplasty (mo)	8.9±5.3	7.4±3.5	0.297
Previously failed urethroplasty (*n*, %)	5 (41.7)	12 (33.3)	0.601
Smoking history (*n*, %)	5 (41.7)	16 (44.4)	0.867
Diabetes (*n*, %)	1 (8.3)	2 (5.6)	0.730
The proximal end of urethra is higher than the pubic ramus based on urethrogram (*n*, %)	7 (58.3)	19 (52.8)	0.738
Stricture length (cm)	3.7±1.1	3.1±0.9	0.077
Follow-up (mo)	36.3±9.5	40.3±7.4	0.137
Results			
Operation time (min)	117.5±27.0	127.1±26.1	0.281
Postoperative fever>38.5°C (*n*, %)	1 (8.3)	4 (11.1)	1.000
Postoperative complications (*n*, %)	0 (0)	2 (5.6)	0.404
Success rate (n/N, %)	11/12 (91.7)	23/36 (63.9)	0.067

TAU, transperineal anastomotic urethroplasty.

### Preoperative evaluation and preparation

A standard evaluation has been performed in all patients, including medical history assessment, physical examination, retrograde, and voiding cystourethrography. For the antibiotic prophylaxis and perineum soaking, it can be referred to our previously published article^9,10^. Anastomotic posterior urethroplasty was performed at least 3 months after pelvic injury or the last failed intervention to ensure the initial healing of extravasation and hematoma resolution.

### Surgical procedure for modified TAU

**Figure 1:**
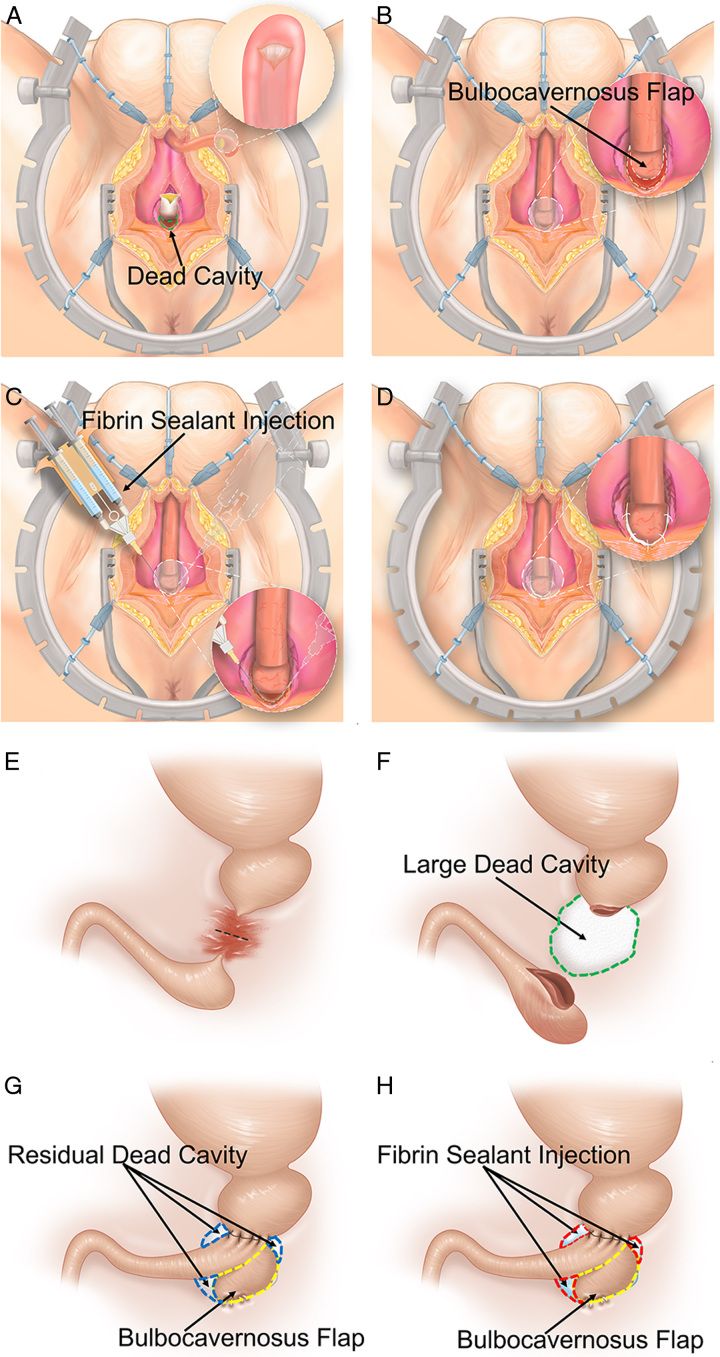
Front view (A–D) and simulated sagittal view (E–H): (A) The urethra was transected at the stricture site and surrounding scar of prostatic apex was resected, which leads to a large dead space (in green dash line); (B) The bulbocavernosus flap (BF) is used to fill the dead space; (C) The human fibrin sealant is injected to fill the dead space; (D) Four to five stitches are sewed on the edge to seal the human fibrin sealant inside; (E) The urethra was transected at the stricture site; (F) a potential large dead cavity (in green dash line) was formed after resecting the surrounding scar; (G) BF (in yellow dash line) was used to fill the large dead cavity. However, there was still residual dead cavity (in blue dash line) existing; and (H) fibrin sealant injection (in red dash line) to fill the residual dead cavity.

After placing the patients in lithotomy position, a perineal midline vertical incision was made overlying the stricture site since our prior study proved that this method is a safer approach than the inverted-U incision for posterior urethroplasty^10^. The bulbospongiosus muscle was incised in the midline. Under the guidance of a metallic sound, the urethra is transected at the distal limit of the stricture (Fig. 1A and E). Then completely resecting the surrounding scar tissue (Fig. 1A and F). Bulbocavernosus flap, which is derived from the posterior swelling segment of corpus cavernosum urethrae, was conventionally resected for following anastomosis. However, due to its good blood supply and adjacent position, bulbocavernosus flap was preserved for the interposition material in our study.

**Figure 2:**
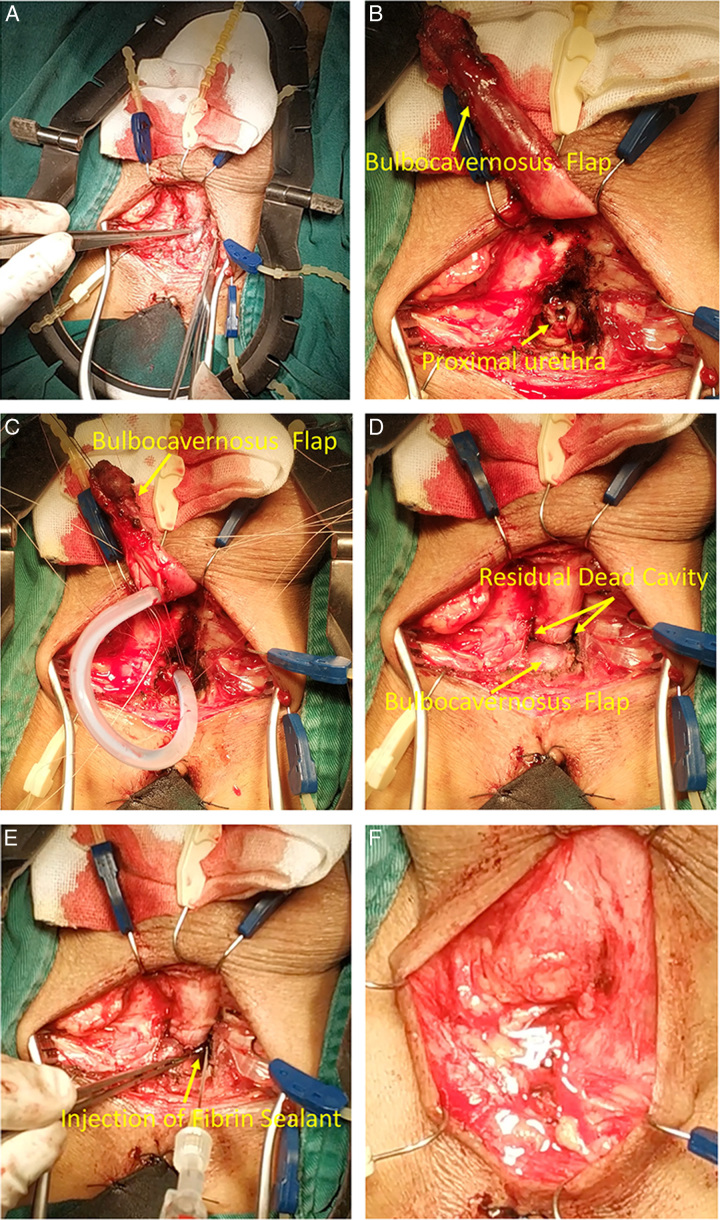
Front view of real surgery: (A) An overview of the surgical site; (B) The urethra is transected at the level of the stricture and surrounding scar tissue is completely removed; (C) The urethral mucosa at both ends were everted to ‘fish-mouth’ like appearance for subsequent valgus mucosa anastomosis. Pay attention to keep the bulbocavernosus flap (BF); (D) BF is interposed to the dead space, but there is still residual dead cavity around the anastomosis that cannot be sutured; (E) The human fibrin glue is injected and filled the dead space; (F) The bulbospongiosus muscle was closed and the incision was closed lay-by-layer.

Before the anastomosis, the urethra mucosa at both ends were everted to ʽfish mouth-ʼ like appearance. Anastomosis was done by 8–10 sutures with 5/0 absorbable suture. After that, we used the bulbocavernosus flap to fill the dead space around the anastomosis (Fig. 1B and G), followed by 2 ml human-plasma-derived fibrin sealant (Shanghai RAAS Blood Products Co, Ltd.) injection (Fig. 1C and H). These two techniques can complement each other to fill the space after scar tissue excision. Next, four to five stitches are sewed on the edge (Fig. 1D). The bulbospongiosus muscle was then closed and the incision was closed lay-by-layer. Finally, pressure dressing was conducted. The real surgery images were shown in Figure 2.

### Surgical procedure for conventional TAU

Conventional TAU meant that bulbocavernosus flap interposition and human-plasma-derived fibrin sealant were not used in the above-mentioned procedures, and the rest of the steps are the same.

### Postoperative management

Patients have dressing change on the second and fourth day postoperatively. Urethral catheter was removed 6–8 weeks after surgery, at which time all the patients were evaluated with retrograde urethrography (RUG) or voiding cystourethrography (VCUG).

### Follow-up

Follow-up methods include regular outpatient visits and telephone follow-up. The criteria for success are that patients remained free of all obstructive symptoms and no further intervention was required. Urethroplasty was regarded failed if the stricture remained, or stricture recurrence developed needing further intervention. Postoperative complications including incision split, incision bleeding, postoperative urethral fistula, and postoperative urinary incontinence were also inquired during the follow-up. Mean follow-up time was 36.3 months for group A and 40.3 months for group B.

### Statistical analysis

Continuous variables are described as mean±SD and compared with one-way ANOVA. Categorical variables are showed as frequencies (%) and compared using χ^2^ or Fisher’s exact tests. *P*<0.05 was considered statistically significant.

## Results

Seventy-one patients with complicated posterior urethral stricture meet the inclusion criteria of our study. After the 1:3 nearest-neighbor propensity score analysis, 48 patients were enrolled in the final analysis, with 12 in the group A and 36 in the group B. The baseline characteristics were presented in the Table 1 and all of them were comparable between these two groups.

**Figure 3:**
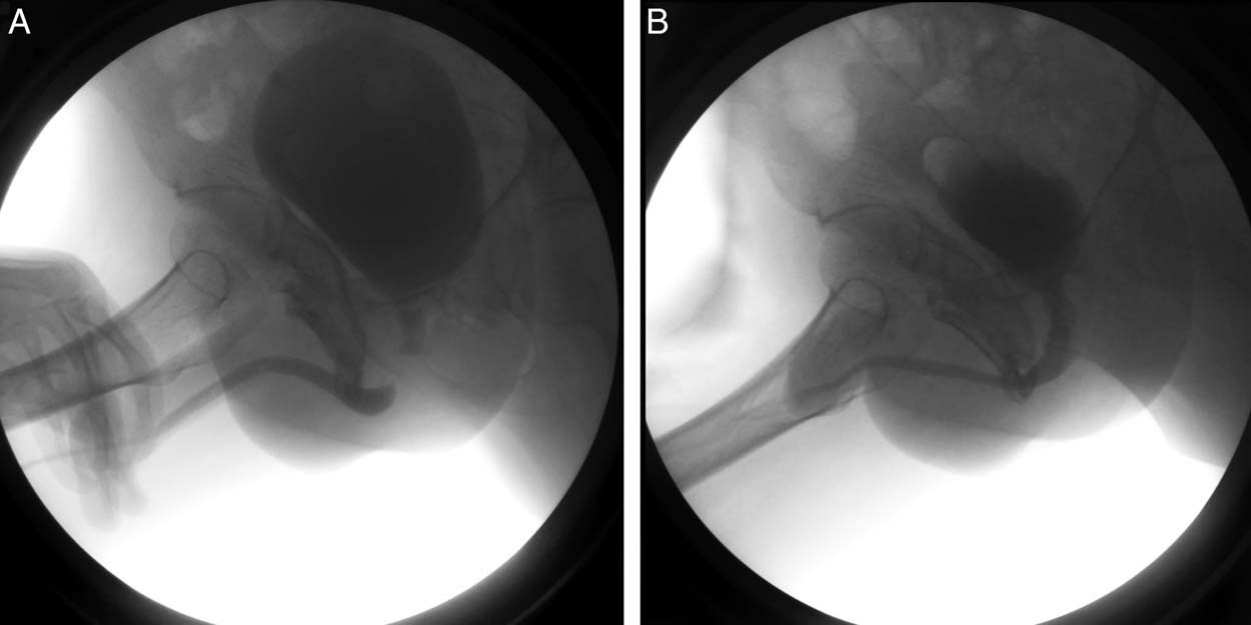
Urethrography: (A) Combined RUG and VCUG preoperatively; (B) VCUG 6–8 weeks after surgery.

The mean operative time was 117.5±27.0 min in group A and 127.1±26.1 min in group B (*P*=0.281). The patients in group A had a higher surgery success rate compared to the patients in group B (91.7 vs. 63.9%, *P*=0.067), with a quasi-significant result. Besides, no postoperative complications were observed in group A, while two patients in group B had postoperative urinary incontinence, but the difference was not significant (0 vs. 5.6%, *P*=0.404). There was no significant difference in the postoperative fever rate (8.3 vs. 11.1%, *P*=1.000) between the two groups. In all patients, postoperative cystourethrography was performed 6–8 weeks after surgery and no urinary leakage was present (Fig. 3).

## Discussion

Currently, TAU is the primary recommended treatment of posterior urethral strictures. However, this surgical approach is not perfect enough especially for complicated cases with long-segment stricture or previously failed surgery since the dead space after the scar tissue excision might lead to a higher postoperative complication rate and recurrence. In our research, we reported a modified TAU combining the bulbocavernosus flap interposition and human fibrin sealant injection to eliminate the dead space, which could improve the surgery success rate compared with conventional TAU.

One of the keys to the successful urethroplasty is complete excision of scarred tissue^11^. However, in complicated cases, this might require removal of a vast amount of tissue and creates a large dead space^12^. The large dead space might lead to blood and exudate accumulation, hematoma formation, or infection^13^. Therefore, two techniques, including bulbocavernosus flap and fibrin sealant, were used to fill the dead space and improve the success rate in our study. Bulbocavernosus flap is derived from the posterior swelling of the corpus spongiosum, which has been used in the repair of rectourethral^14^. However, to the best of our knowledge, there have been no reported cases applying bulbocavernosus flap for urethroplasty. In our opinion, bulbocavernosus flap can be an ideal interposition material during posterior urethroplasty because of its adjacent position and good supply^14^. After complete resection of the stricture and surrounding scars, it can be retained to serve as an extra protective cover for the anastomosis and fill the dead space. Additionally, it can also serve as a bottle cap for the dead space, firmly sealing the fibrin glue into the dead space. All these features make bulbocavernosus flap being a natural and excellent interposition material during the posterior urethroplasty.

In addition to bulbocavernosus flap, we also used the human-plasma-derived fibrin sealant, namely Fibingluraas (Shanghai RAAS Blood Products Co, Ltd.) in our research simultaneously. Although bulbocavernosus flap has been applied to fill the dead space after scar tissue excision, it could be inferred that there might still be some potential dead space that cannot be filled completely. In this condition, we further used the fibrin sealant to compensate this shortcoming. This fibrin sealant is mainly made up of two components, including fibrinogen and thrombin, and thereby will promote the final stage of the coagulation cascade. In addition to hemostasis, it has also been reported that fibrin sealant could promote tissue adhesion^15^, decrease inflammation^16^, and enhance re-endothelization^17^. All these features could reinforce the anastomotic suture line^18^ and accelerate the healing. Moreover, unlike synthetic tissue adhesive agents, it is human-derived and therefore biocompatible and biodegradable. Therefore, we believe it could be an ideal adjunct for urethral reconstruction surgeries. Application of fibrin sealant in urethroplasty has been reported in several studies^17,19,20^ previously. In the study conducted by Barbagli^19,20^, six patients underwent bulbar urethroplasty with fibrin glue. During a mean follow-up period of 16 months, no repeat strictures were demonstrated in any patient, indicating that fibrin sealant had promising prospects. However, it is worth noting that the follow-up duration in their study was relatively short and the absence of a control group makes it challenging to evaluate the efficacy of fibrin sealant conclusively. In another study, Hick^17^ found that fibrin sealant could enhance wound healing in penile urethroplasty. However, no follow-up data was reported in their study.

In summary, complicated posterior urethroplasty remains a challenge for urologists. Here, we reported a modified TAU combining bulbocavernosus flap interposition and human-plasma-derived fibrin sealant, which could improve the surgery success rate compared with conventional TAU. We believe this method is optimal for complicated posterior urethral stricture and might improve the prognosis. Nevertheless, our study has several limitations. First, the sample size of our study was relatively small and we only observed a quasi-significant result in our research. Further well-designed prospective trials with a larger sample size are necessary to obtain conclusive results.

Second, due to the three-dimensional irregularity of the dead space, we were unable to measure its size. Future studies that include predictive imaging to estimate the size of dead space should be conducted^12^.

## Conclusion

Based on the preliminary results, modified TAU with bulbocavernosus flap interposition and the use of human fibrin sealant could improve the success rate in complicated posterior urethral stricture reconstruction. Further well-designed prospective trials with a larger sample size are necessary to obtain conclusive results.

## Ethical approval

Ethical approval for this study was provided by the West China Hospital of Sichuan University Medical Research Ethics Committee (Reference number: 2022737).

## Consent

Informed consent is waived in this study, since this is a retrospective study and no identifying details are reported.

## Sources of funding

This work was supported by 1.3.5 project for disciplines of excellence, West China Hospital, Sichuan University (grant no. ZY2016104).

## Author contribution

M.W., L.Z., and K.W.: conception and design of the study; M.W., L.Z., Y., M., J., Y., J., and L.: acquisition of data; M.W., Z., and L.: data analysis and/or interpretation; M.W., Z., L., Y., M., J., and Y.: drafting of the manuscript; J., L., and K.W.: critical revision of the manuscript for important intellectual content; M.W., Z., and L.: statistical analysis; K.W.: obtaining funding. All authors have read and approved the manuscript.

## Conflicts of interest disclosure

There are no conflicts of interest.

## Research registration unique identifying number (UIN)

1. Name of registry: research registry.
2. Unique identifying number: researchregistry9356.
3. Hyperlink: https://www.researchregistry.com/browse-theregistry#home/registrationdetails/64ca3db7dc84490028807e0e/

## Guarantor

Kunjie Wang.

## Data sharing statement

Data are available for researchers who request it from the authors.

## Provenance and peer review

No.
